# Exploring drug sensitivity guided individualized treatment in pediatric spindle cell/sclerosing rhabdomyosarcoma

**DOI:** 10.3389/fphar.2026.1924792

**Published:** 2026-08-07

**Authors:** Yi-Tian Chang, Yuan Zhang, Yu-Tong Zhang

**Affiliations:** 1 Department of Pediatric Oncology, Children’s Medical Center, The First Hospital of Jilin University, Changchun, Jilin, China; 2 Department of Ophthalmology, The Third People’s Hospital Affiliated to Dalian Medical University, LiaoNing, China

**Keywords:** drug sensitivity assay, pediatric, personalized therapy, rhabdomyosarcoma, spindle cell/sclerosing rhabdomyosarcoma

## Abstract

**Purpose:**

Spindle cell and sclerosing rhabdomyosarcoma (sRMS/scRMS) are rare variants accounting for 5%–10% of all RMS cases. This study aimed to evaluate the feasibility of using drug sensitivity assay to guide real time treatment decisions in children with sRMS/scRMS.

**Methods:**

We retrospectively analyzed 14 consecutive patients diagnosed with sRMS/scRMS between January 2020 and December 2024. All patients initially received two cycles of VAC (vincristine/dactinomycin/cyclophosphamide). If the tumor response is stable or progressive, subsequent treatment is tailored based on drug sensitivity assay. The primary endpoint was feasibility of drug sensitivity assay guided decision making. Secondary endpoints included objective response rate (ORR), PFS, and OS.

**Results:**

Drug sensitivity assay revealed uniform platinum sensitivity across all patients. After two cycles of VAC, 13 of 14 patients exhibited either stable or progressive disease, suggesting limited efficacy of the VAC regimen in this cohort. These 13 patients were subsequently transitioned to a platinum containing regimen guided by drug sensitivity assay, following which an objective response was observed in all cases. The 5-year PFS and OS were 36.92% (95% CI, 11.82–61.33) and 53.85% (95% CI, 31.70–82.93), respectively. Compared with the historical median survival of 9 months for relapsed/refractory RMS, the outcomes in our cohort appeared to be somewhat longer, suggesting a potential clinical benefit. The approach proved to be feasible in all 14 patients.

**Conclusion:**

Our findings suggest that drug sensitivity assay may represent a feasible and potentially useful tool for informing real time treatment decisions in children with sRMS/scRMS. The incorporation of such testing into the therapeutic algorithm for this chemotherapy resistant sarcoma variant may offer a means to individualize treatment and possibly improve prognostic outcomes. However, given the exploratory nature and limited sample size of this study, these observations should be interpreted with caution, and larger prospective studies are needed to confirm their validity.

## Introduction

Spindle cell and sclerosing rhabdomyosarcoma (sRMS/scRMS), which accounts for 5%–10% of all RMS, was reclassified as a distinct pathologic entity in the latest world health organization classification of soft tissue tumors one decay ago (Owosho et al., 2016; Sbaraglia et al., 2021; Dehner et al., 2024; De Vita et al., 2021). SRMS/scRMS was first described by the German Italian Cooperative Sarcoma Study on the basis of its distinct clinicopathologic features and favorable outcome, resulting in separation from the more common embryonal RMS (ERMS) (Cavazzana et al., 1992). Consequently, both the Children’s Oncology Group (COG) and the European pediatric Soft tissue sarcoma Study Group (EpSSG) classify sRMS/scRMS as a favorable histologic subtype and therefore do not include it as a factor affecting risk group stratification for RMS (Bi et al., 2019; Borinstein et al., 2018). Due to the rarety of this type of RMS, current reports on sRMS/scRMS consist predominantly of case analyses, and the prognosis is generally regarded as favorable (Whittle et al., 2019; Arja et al., 2026). However, some studies indicate that sRMS/scRMS arising in the head and neck region, particularly in patients harboring MYOD1 mutations is associated with a poorer prognosis (Poojari and Madabhavi, 2025; Agaram et al., 2014; Ahmed et al., 2021; De Vita et al., 2022). In our clinical experience with a number of sRMS/scRMS cases, we have observed that this subtype appears to be less chemosensitive than embryonal RMS. Most sRMS/scRMS tumors show limited responses to chemotherapy, and even the minority that achieve an initial response tend to relapse rapidly. These observations prompted us to question whether sRMS/scRMS truly confers a favorable prognosis, and whether the current risk-based treatment stratification for RMS is genuinely applicable to this subtype. To explore these questions, we conducted a retrospective analysis of sRMS/scRMS cases diagnosed at our institution, reviewing their biological characteristics, treatment approaches, therapeutic responses, and survival outcomes, with the aim of sharing our experience in individualized treatment strategies incorporating drug sensitivity assay.

## Methods

Medical records of patients diagnosed with sRMS/scRMS between 1 January 2020, and 31 December 2024, were reviewed. The collection of clinical records was approved by the Institutional Review Board of The First Hospital of Jilin University. Informed consent was obtained from the parents or legal guardians of all patients. The following data were extracted for each patient: age, sex, histological subtype, tumor stage, risk group, biomarkers, gene expression, chemotherapy regimen and dosage, surgical details, radiation details, treatment outcomes, treatment toxicity and follow up.

The diagnosis of RMS is typically established through tissue examination using conventional histology, which may be supplemented by immunohistochemistry (Ferrari et al., 2022). After the diagnosis of RMS, whole body lymph node ultrasound, chest CT, abdominal CT, brain MRI, and whole body [^18^F]FDG-PET/CT are mandatory. At least unilateral biopsies for morphologic assessment of bone marrow tumor were performed. Molecular testing using next-generation sequencing (NGS) for both DNA and RNA is mandatory. The stageing and risk stratification were assessed according to criteria using the Intergroup Rhabdomyosarcoma Study grouping (Borinstein et al., 2018). Brifely, the risk stratification for RMS is divided into low, intermediate, and high categories. Low risk applies only to fusion negative (FN) RMS and includes Group I, Stage 1 and 2; Group II, Stage 1 and 2; and Group III, Stage 1 orbit. Intermediate risk (applicable to any fusion status) includes: any stage of Group I/II/III fusion positive (FP) RMS; Group I/II, Stage 3 F N-RMS; any stage of Group III FN-RMS (except orbit); and Group IV, Stage 4 F N-RMS in patients younger than 10 years. High risk (any fusion status) includes: Group IV, Stage 4 F N-RMS in patients older than 10 years, and Group IV, Stage 4 F P-RMS.

The drug sensitivity assay was conducted on peripheral blood samples obtained from cancer patients, employing the Sequenom single nucleotide polymorphism (SNP) mass spectrometry platform in conjunction with matrix-assisted laser desorption/ionization time-of-flight mass spectrometry (MALDI-TOF MS) (Ledesma et al., 2023). Briefly, MALDI-TOF MS-based genotyping of peripheral blood constitutes a high throughput molecular diagnostic strategy for interrogating SNPs in genes implicated in chemotherapeutic drug response. The workflow commences with genomic DNA extraction from ethylenediaminetetraacetic acid anticoagulated peripheral blood, followed by multiplex Polymerase Chain Reaction (PCR) to simultaneously amplify DNA fragments harboring the target SNP loci. The PCR products are then treated with shrimp alkaline phosphatase to remove residual deoxynucleotide triphosphates, after which a single base extension reaction is performed using locus specific primers. This reaction incorporates exactly one nucleotide at each SNP site, yielding extension products that differ in mass by a single base. Following purification, the extension products are co-crystallized with a matrix and spotted onto a chip for MALDI-TOF MS analysis. Upon pulsed laser irradiation, the analytes are desorbed and ionized, and the resulting singly charged ions are separated in a time-of-flight mass analyzer according to their mass-to-charge ratio, thereby enabling precise resolution of single base mass differences. Genotypes at each SNP locus are subsequently assigned using dedicated data analysis software. By synergistically integrating the multiplexing capacity of PCR with the high resolution of mass spectrometry, this technology allows parallel genotyping of multiple chemotherapy related loci in a single analytical run. Accordingly, it offers clinically valuable guidance for the individualized selection of commonly used anticancer agents, including platinum compounds, taxanes, anthracyclines, fluoropyrimidines, and alkylating agents. Results of the drug sensitivity assay were typically available approximately 2 weeks after blood collection.

Any patient who received at least one dose of therapy after drug sensitivity assay was eligible for response assessment. The tumor response was assessed using the Response Evaluation Criteria in Solid Tumors (RECIST) version 1.1. Complete response (CR) was defined as the disappearance of all target lesions, and partial response (PR) as at least a 30% decrease in the sum of the diameters of target lesions from baseline. Progressive disease (PD) was defined as at least a 20% increase in the sum of the diameters of target lesions relative to the smallest sum recorded during treatment, with an absolute increase of at least 5 mm, or the appearance of one or more new lesions or unequivocal progression of non-target lesions. Stable disease (SD) was defined as neither sufficient shrinkage to qualify for PR nor sufficient increase to qualify for PD (Eisenhauer et al., 2009). Tumor disease evaluations were performed every other cycle while on therapy. For documentation of objective response, confirmatory imaging was required to be obtained following the next consecutive cycle for any patient with CR or partial response PR.

## Statistical analysis

The primary objective of this study was to evaluate the feasibility of using peripheral blood based drug sensitivity assay to guide real time treatment decisions in children with chemotherapy resistant sRMS/scRMS. The secondary objectives were to assess the safety of individualized treatment plans and to evaluate treatment activity based on overall response rate (ORR), progression free survival (PFS), and overall survival (OS). Given the exploratory nature and limited sample size of this single center retrospective cohort (n = 14), multivariable analysis and sensitivity analyses were not performed, as the cohort size precluded meaningful adjustment for multiple covariates. Accordingly, the results should be interpreted as descriptive and hypothesis generating.

PFS and OS were determined using the Kaplan-Meier method with Rothman’s 95% confidence interval (CI). PFS was defined as the date of beginning chemotherapy until the first occurrence of disease progression or relapse. OS is defined as the date of beginning chemotherapy to the date of death due to any cause. These analyses were performed with Prism 10.

## Results

Between 1 January 2020, and 31 December 2024, a total of 14 sRMS/scRMS patients were diagnosed. The clinical characteristics of these patients are listed in Table 1.

**TABLE 1:** clinical characteristics of the fourteen patients.

Patient	Age at diagnosis (years)/sex	Primary tumor site	Site	Group	Stage	Risk stratification	The maximum diameter of the tumor	Lymphonodes	Metastasis diseases	Gene testing
1	13/M	Nasopharynx	Unfavorable	III	3	High	4.5 cm	N1	M-	Fusion (−)
2	12.5/F	Limbs	Unfavorable	IV	4	High	8 cm	N1	M+	Fusion (−)
3	13/M	Limbs	Unfavorable	IV	4	High	8 cm	N1	M+	Fusion (−)
4	8/M	Pelvic	Unfavorable	IV	4	Intermediate	11 cm	N1	M+	Fusion (−)
5	9/F	Limbs	Unfavorable	IV	4	Intermediate	7 cm	N1	M+	Fusion (−)
6	6/M	Perimeningeal	Unfavorable	IV	4	Intermediate	5.5 cm	N1	M-	MYOD1 mutation
7	3.5/M	Limbs	Unfavorable	IV	4	Intermediate	7 cm	N1	M-	MYOD1 mutation
8	11/M	Perimeningeal	Unfavorable	III	3	High	6 cm	N1	M+	Fusion (−)
9	8/M	Limbs	Unfavorable	IV	4	Intermediate	7 cm	N1	M-	Fusion (−)
10	14/F	Pelvic	Unfavorable	III	3	High	8 cm	N1	M+	Fusion (−)
11	11/M	Nasopharynx	Unfavorable	III	3	High	3 cm	N1	M+	Fusion (−)
12	7/M	Pelvic	Unfavorable	III	3	Intermediate	6 cm	N1	M-	Fusion (−)
13	10/M	Limbs	Unfavorable	IV	4	High	12 cm	N1	M+	Fusion (−)
14	13/F	Limbs	Unfavorable	IV	4	High	10 cm	N1	M+	Fusion (−)

Abbreviations: F, female; M, male; N, lymph node; M, metastasis; fusion PAX3/7-FOXO1-fusion.

There were 10 males and four females, with age at diagnosis ranging from 3.5 to 13 years (mean: 9.85 years). The anatomic sites included the extremity (7 cases), pelvic and buttock muscles (3 cases), nasopharynx (2 cases), and soft tissue of the skull (2 cases). Except for the 10 children with limb and pelvic tumors, the remaining 4 cases of head and neck tumors were all considered parameningeal sites. Tumor size ranged from 4.5 to 12 cm, with 12 cases measuring >5 cm in greatest dimension. Risk stratification was assessed according to the IRS grouping staging criteria as previously described. Six patients were classified as intermediate risk, while the remaining eight patients were high risk. All patients underwent NGS of RNA and DNA for RMS, in addition to drug sensitivity assay.

Among the 14 patients, two younger patients with intermediate risk group were found to harbor MYOD1 gene mutations (Hettmer et al., 2022), whereas the remaining patients lacked the FOXO1 fusion gene and any other meaningful genetic abnormalities. All patients, regardless of intermediate or high-risk status, initiated treatment with the VAC chemotherapy regimen, which consisted of vincristine 1.5 mg/m^2^ on days 1, 8, and 15; dactinomycin 0.045 mg/(kgdose) in normal saline as a 5-min intravenously infusion (IV) on day 1; cyclophosphamide 1.2 g/m^2^ as a 1-h IV on day 1, with mesna 360 mg/(m^2^·dose) in normal saline given as a 20–30 min IV at 0, 3, 6, and 9 h. After two cycles of VAC, all patients had stable disease in response to treatment except Patient No. 12. Once genetic testing and drug sensitivity assay results became available, the chemotherapy regimen was adjusted to include agents to which the tumors were sensitive, as confirmed by drug susceptibility testing. All patients, including No. 12, were found to be sensitive to platinum-based drugs, while No. 12 was also sensitive to etoposide and cyclophosphamide. Considering patient No. 12s good response to initial VAC therapy, his sensitivity to cyclophosphamide shown by drug sensitivity testing, and his intermediate risk status, we continued the VAC regimen for a total of 12 cycles once every 3 weeks, for a total duration of 42 weeks without adjusting his treatment plan. He maintained CR throughout treatment and remained disease free at 42 months of follow up. The detailed information of treatment response and outcome of these patients was shown in Table 2.

**TABLE 2:** The detailed information of treatment response and outcome.

Patient	Age at diagnosis (years)/sex	Treatment before drug screening test	Treatmment response before drug screening test	Drug screening test	Treatment response after drug screening test	Surgery	Radiation therapy	Follow up (months)	Outcome
1	13/M	VAC×2+VI	PD	Platinum sensitive	PR	R1	Y	49	CR
2	12.5/F	VAC/VI/VDC/I.E.,	PD	Platinum sensitive	PR	R1	Y	41	CR
3	13/M	VAC×2	PD	Platinum sensitive	PR	R1	Y	21	DOD
4	8/M	VAC×2	SD	Platinum sensitive	PR	R1	Y	38	CR
5	9/F	VAC×2	SD	Platinum sensitive	CR	R0	Y	33	CR
6	6/M	VAC×2	SD	Platinum sensitive	CR	R0	Y	12	DOD
7	3.5/M	VAC×2	SD	Platinum sensitive	CR	R0	Y	9	DOD
8	11/M	VAC×2	SD	Platinum sensitive	CR	R0	Y	37	RELAPSED
9	8/M	VAC×2	SD	Platinum sensitive	CR	R0	Y	45	CR
10	14/F	VAC×2	SD	Platinum sensitive	CR	R0	Y	28	RELAPSED
11	11/M	VAC×2	SD	Platinum sensitive	CR	R0	Y	14	DOD
12	7/M	VAC×2	PR	Platinum/etoposide/cyclophosphamide sensitive	CR	R0	Y	42	CR
13	10/M	VAC×2	SD	Platinum sensitive	PR	R1	Y	9	DOD
14	13/F	VAC×2	PD	Platinum sensitive	PR	R1	Y	21	DOD

Abbreviations: VAC, vincristine/dactinomycin/cyclophosphamide; VI, vincristine/irinotecan; VDC, vincristine/doxorubicin/cyclophosphamide; I.E., (ifosfamide/etoposide); PD, progression disease; SD, stable disease; PR, partial response; Y yes; CR, complete response; DOD, die of disease.

The remaining 13 patients were switched to the CEV (carboplatin/epirubicin/vincristine) regimen. This regimen was selected because drug sensitivity assay indicated platinum sensitivity in these patients, and CEV contains carboplatin (Frascella et al., 1996). Furthermore, the CEV regimen had demonstrated the highest objective response rate among second line treatments for relapsed RMS (Winter et al., 2015). The CEV regimen comprised carboplatin 500 mg/m^2^ IV over 1 h on day 1, epirubicin 150 mg/m^2^ IV over 6 h on day 2, and vincristine 1.5 mg/m^2^ (max 2 mg) as an IV bolus on days 1, 8, and 15. The original protocol for RMS typically alternates the CEV regimen with VIE (ifosfamide, vincristine, and etoposide), limiting CEV to no more than three cycles. Nevertheless, given that drug sensitivity assay in our cohort consistently showed platinum sensitivity and that the safe cumulative dose of epirubicin is 900 mg/m^2^ (Ryberg et al., 2008), each patient received six cycles of CEV. Following these six cycles, the regimen was changed to carboplatin, etoposide, and vincristine, which consisted of vincristine 1.5 mg/m^2^ (max 2 mg) on days 1, 8, and 15; carboplatin 500 mg/m2 on day 1; and etoposide 150 mg/m^2^ for 3 days (Oberlin et al., 2012). The high risk group received nine cycles of carboplatin/etoposide/vincristine, while the intermediate risk group received six cycles.

Following the regimen adjustment, among the eight high risk patients, five achieved PR and subsequently underwent R1 resection, while three achieved CR and underwent R0 resection. Among the six intermediate risk patients, two achieved a PR; of these, one underwent R0 resection and the other underwent R1 resection. The remaining three intermediate risk patients, including patient No. 12, achieved complete response, all of whom underwent R0 resection. All patients received radiotherapy to the primary tumor and any residual lesions. All high risk patients received maintenance therapy with vinorelbine plus cyclophosphamide after completion of treatment (Bi et al., 2019). Excluding patient No. 12, the remaining 13 patients, six died and two experienced relapse. The 5-year PFS was 36.92% (95% CI, 11.82–61.33), median PFS was 28months And the 5-year OS was 53.85% (95% CI, 31.70–82.93).

All toxicity were collected and graded based on the National Cancer Institute Common Terminology Criteria for Adverse Events, version 4.0 (Miller et al., 2019). All 14 patients were evaluable for toxicity. The most common grade 3 or four adverse events were hematological, including neutropenia (12/14, 85.7%), thrombocytopenia (10/14, 71.4%), and anemia (14/14, 100%). Febrile neutropenia occurred in five patients (35.7%), all of whom recovered with antibiotic support and granulocyte colony stimulating factor administration. Non hematological toxicities included grade 2 to 3 nausea and vomiting (9/14, 64.3%), grade 1 to 2 mucositis (6/14, 42.9%), and transient transaminase elevation (4/14, 28.6%). No patient developed clinically significant nephrotoxicity or ototoxicity during the treatment period. Regarding anthracycline exposure, the cumulative epirubicin dose was 900 mg/m^2^ in all patients who completed six cycles of CEV, which remained within the recommended lifetime limit. No clinical evidence of cardiotoxicity was observed during follow-up. One patient experienced grade 2 peripheral neuropathy attributed to vincristine, which partially resolved after dose reduction. There were no treatment related deaths.

## Discussion

Consensus has not been reached as to the optimal type of treatment of sRMS/scRMS (Robinson et al., 2013). Previously, in the treatment of RMS, we selected regimens based on risk group assignment.

According to the last 2005 EpSSG RMS stratification, first line chemotherapy includes vincristine and actinomycin (VA) for the low risk group, adding ifosfamide (IVA) for standard and high risk groups and doxorubicin for the very high risk group (IVADo) (De Salvo et al., 2024; Bisogno et al., 2018; Ben-Arush et al., 2022). This multimodal therapy has shown overall survival rates over 90% in patients with a low risk localized disease, but only of 21% or 30% in patients with a metastatic or recurrent disease, respectively (Terwisscha van Scheltinga et al., 2022; Bisogno et al., 2025). Our department utilizes the U.S. consensus risk stratification strategy for treating RMS. For low risk RMS, the preferred regimen is four cycles of VAC followed by local control and four cycles of VA (vincristine and dactinomycin); an alternative is four cycles of VA followed by twelve cycles of VAC. For intermediate risk RMS, the preferred approach alternates VAC and VI (vincristine and irinotecan): VAC × 3 and VI × 2, then alternating VAC/VI with local control, totaling VAC × 4 and VI × 5. An alternative for intermediate risk RMS is VDC (vincristine, doxorubicin, and cyclophosphamide) with VAC × 4 followed by local control and VAC × 8. For high risk RMS, a reasonable and preferred strategy uses multiple agents:VAC, VAC/VI, VDC, and, I.E., (ifosfamide and etoposide) over 51 weeks, with weekly vincristine given on alternating weeks and local control integrated (Borinstein et al., 2018; Haduong et al., 2022). There is an emerging trend toward incorporating molecular markers into therapy and recurrence prediction, with the goal of providing patients with different treatment options (Hettmer et al., 2022). However, in clinical practice, we encountered three cases of sclerosing rhabdomyosarcoma in the high risk group that showed disease progression after two to three cycles of the high risk regimen. Among them, Case 1 had a nasopharyngeal lesion that progressed despite chemotherapy, leading to dyspnea that required surgical intervention to relieve airway obstruction. The tumor was not completely resected and recurred at the nasopharynx approximately 3 weeks later, accompanied by the development of bilateral lung metastases. The parents were strongly motivated to continue treatment. Based on drug screening testing reports indicating platinum sensitivity, a second line regimen consisting of epirubicin, carboplatin, and vincristine was selected. After one cycle, the child’s nasal mass was observed to shrink and dyspnea improved. After two cycles, evaluation showed PR of both the nasopharyngeal primary tumor and the lung metastases. After six additional cycles of the second line regimen, evaluation revealed a complete response of both the primary tumor and lung metastases. Due to concerns about cumulative anthracycline dose, the second line regimen was changed to carboplatin, etoposide, and vincristine for nine cycles, followed by maintaince chemotherapy. He maintained CR throughout treatment and remained disease free at 49 months of follow up.

sRMS/scRMS type is a rare variant of RMS, accounting for only 5%–10% of RMS cases and was first described in 1992 as a prognostically favorable variant of embryonal RMS in children (Cavazzana et al., 1992; Yasui et al., 2015). Previous limited case studies suggest that the prognosis of sRMS/scRMS in the pediatric population is favorable and initially show good response to VAC (Leuschner et al., 1993), but >50% of tumors recur or progress, which suggest a worse prognosis of sRMS/scRMS compared to the pediatric embryonal variant.

In our cohort of patients with s sRMS/scRMS, nearly all patients were insensitive to the VAC regimen. Only one patient achieved a PR after the second cycle of VAC, while the remaining patients had SD or PD, indicating that sRMS/scRMS is generally not sensitive to VAC. Selecting chemotherapy based solely on risk group classification therefore appears suboptimal, as regimen choice is critical for these patients and directly impacts prognosis. After the first two patients, who received risk based VAC chemotherapy, developed disease progression, we discontinued the high risk regimen and switched to a platinum containing second line regimen guided by drug sensitivity testing. As expected, tumor regression was observed following the addition of the sensitive drugs. To ensure that an insufficient number of VAC cycles did not confound the assessment of its true efficacy, we deliberately administered two cycles of VAC to all patients. Only after confirming that the VAC regimen was indeed insensitive did we adjust treatment based on drug sensitivity testing results.

A total of 14 patients were included in this cohort. Except for one patient whose regimen remained unchanged based on both VAC response and drug screening testing, the remaining 13 patients exhibited insensitivity to the VAC regimen, with response assessed as stable disease or progressive disease after two cycles. Following a regimen switch guided by drug screening testing, the ORR reached 100%. All patients underwent surgery and radiotherapy to the primary tumor, including irradiation of residual lesions. Among these 13 patients, six died, two relapsed but remained alive, and five achieved disease free survival, corresponding to a 5-year PFS of 36.92% and an 5-year OS of 53.85%. Compared with the historical median survival time of 9 months for relapsed/refractory RMS (Casanova et al., 2023), The outcomes observed in our study appear to be somewhat longer, suggesting a potential clinical benefit.

The primary objective of this study was to determine the feasibility of using this process for the treatment of children with sRMS/scRMS. We have shown that this was feasible in all 14 patients. Furthermore, even in refractory sRMS/scRMS, the use of drug screening testing to guide treatment selection has yielded promising survival outcomes.

**FIGURE 1:**
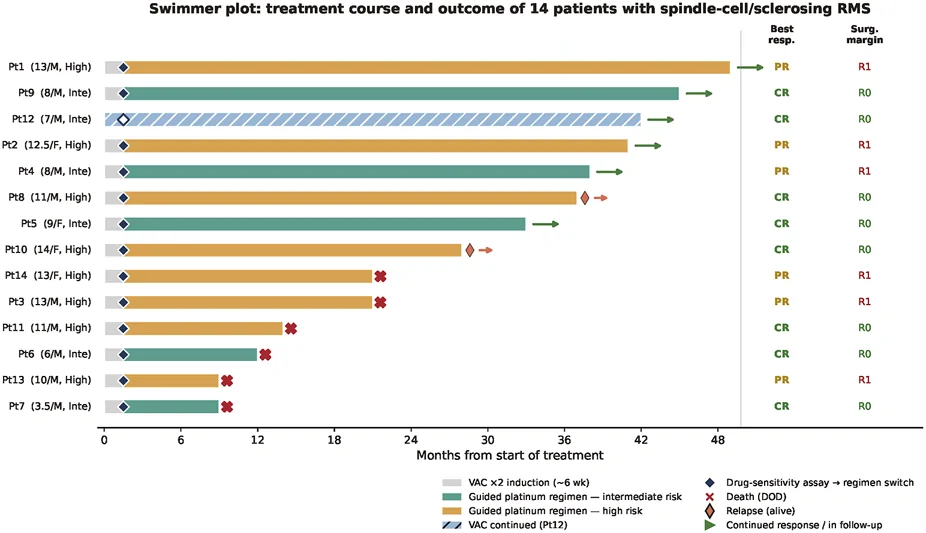
Swimmer plot of the individual treatment course and outcome of the 14 patients with spindle-cell/sclerosing rhabdomyosarcoma. Each horizontal bar represents one patient (labelled with age/sex and risk group) and is ordered by follow-up duration; bar length corresponds to follow-up time in months. The grey segment denotes the initial two cycles of VAC induction (∼6 weeks). The dark blue diamond marks the time point at which the drug-sensitivity assay result became available and the chemotherapy regimen was switched; the open diamond (Patient 12) indicates that the assay was performed but VAC was continued because of an initial response. Teal and amber segments represent the drug sensitivity guided platinum-containing regimen in intermediate- and high-risk patients, respectively. End of bar symbols denote outcome: red cross, death of disease (DOD); orange diamond with arrow, relapse (alive); green arrow, continued response/ongoing follow-up. The two right hand columns summarise the best response to the guided regimen and the surgical resection margin. All 14 patients received surgery and radiotherapy to the primary site. CR, complete response; PR, partial response; R0, microscopically negative margin; R1, microscopically positive margin; VAC, vincristine/dactinomycin/cyclophosphamide.

Although our study has several limitations, namely, the small sample size and the use of peripheral blood rather than tumor algorithm based predictions for drug sensitivity assay, this study demonstrates that peripheral blood drug sensitivity assay is a feasible and clinically valuable approach for guiding real time treatment decisions in children with sRMS/scRMS. The conventional risk based VAC regimen seemed to yield suboptimal responses in this rare subtype, with 13 of 14 patients showing disease progression or stable disease after two cycles. In contrast, switching to a drug sensitivity guided, platinum containing second-line regimen achieved an objective response rate of 100%. A swimmer plot illustrating the treatment course, regimen switches, best response, surgical margin, and outcome for each of the 14 patients is presented in Figure 1.

Currently, drug sensitivity testing for tumor tissues relies heavily on organoid culture, a technique that is both time consuming and not yet commercially available in China (Zhao et al., 2024). To explore alternative approaches, previous studies have investigated the feasibility of substituting peripheral blood lymphocytes or leukocytes for tumor tissues in chemosensitivity assays. In these investigations, 30 commonly used anticancer agents were grouped into five mechanistic categories: inhibitors of nucleic acid synthesis, inhibitors of protein synthesis and function, topoisomerase inhibitors, agents directly targeting DNA structure and function, and transcription inhibitors. These findings indicated that, for the majority of adult cancer types, the correlation and concordance between peripheral blood based and tumor tissue based sensitivity results were favorable (Nakamura et al., 2006; Ashley et al., 2008). At the molecular level, peripheral blood cells and tumor cells exhibited substantial concordance in the expression of multidrug resistance related genes, including MDR1/P-gp, p53, and GST-π, among others, supporting the notion that peripheral blood cells may reasonably substitute for tumor cells in drug sensitivity testing for most cancers (Malik et al., 1997). Although pediatric tumors are generally more chemosensitive, thereby potentially limiting the clinical utility of routine drug sensitivity testing in this population, such assays may still offer individualized therapeutic guidance for the subset of chemotherapy insensitive pediatric tumors, thereby potentially improving the likelihood of cure.

Our study has several limitations, including a small sample size, a single center retrospective design, the use of peripheral blood based SNP genotyping rather than direct tumor cytotoxicity assays, the lack of a contemporaneous control group, and variable follow up durations. Despite these constraints, we found that the conventional risk based VAC regimen yielded suboptimal responses in most sRMS/scRMS patients, whereas switching to a drug sensitivity guided platinum-containing regimen achieved a 100% ORR. While these promising results necessitate cautious interpretation and independent confirmation in larger prospective cohorts, our preliminary data indicate that pharmacogenomic profiling can be feasibly incorporated into clinical decision making and may offer therapeutic benefit for patients with chemotherapy insensitive sRMS/scRMS.

## Data Availability

The original contributions presented in the study are included in the article/supplementary material, further inquiries can be directed to the corresponding author.
